# Calvaria Critical Size Defects Regeneration Using Collagen Membranes to Assess the Osteopromotive Principle: An Animal Study

**DOI:** 10.3390/membranes12050461

**Published:** 2022-04-24

**Authors:** Vinícius Ferreira Bizelli, Edith Umasi Ramos, Allice Santos Cruz Veras, Giovana Rampazzo Teixeira, Leonardo P. Faverani, Ana Paula Farnezi Bassi

**Affiliations:** 1 Department of Diagnosis and Surgery, School of Dentistry, São Paulo State University (UNESP), Rua José Bonifácio, 1193, Araçatuba 16015-050, SP, Brazil; yassed_20@hotmail.com (E.U.R.); leonardo.faverani@unesp.br (L.P.F.); ana.bassi@unesp.br (A.P.F.B.); 2 Multicenter Graduate Program in Physiological Sciences, SBFIS, São Paulo State University (UNESP), Rua Roberto Simonsen, 305, Presidente Prudente 19060-900, SP, Brazil; allice.santos@unesp.br (A.S.C.V.); giovana.rampazzo@unesp.br (G.R.T.)

**Keywords:** membranes, bone regeneration, inflammation

## Abstract

Guided bone regeneration (GBR) is a common practice in implantology, and it is necessary to use membranes in this process. The present study aimed to evaluate the osteopromotive principle of two porcine collagen membranes in critical-size defects at rats calvaria. Ninety-six Albinus Wistar rats were divided into BG (positive control), JS, CS, and CG (negative control) groups and were sacrificed at 7, 15, 30, and 60 days postoperatively. The samples were assessed by histological, histometric, immunohistochemical, and microtomographic analyses. More intense inflammatory profile was seen in the JS and CS groups (*p* < 0.05). At 60 days, the JS group showed a satisfactory osteopromotive behavior compared to BG (*p* = 0.193), while CS did not demonstrate the capacity to promote bone formation. At the immunohistochemical analysis, the CS showed mild labeling for osteocalcin (OC) and osteopontin (OP), the JS demonstrated mild to moderate for OC and OP and the BG demonstrated moderate to intense for OC and OP. The tridimensional analysis found the lowest average for the total volume of newly formed bone in the CS (84,901 mm^2^), compared to the BG (319,834 mm^2^) (*p* < 0.05). We conclude that the different thicknesses and treatment techniques of each membrane may interfere with its biological behavior.

## 1. Introduction

The concept of guided bone regeneration (GBR) consists of the use of a membrane as a barrier, which is associated or not with the use of particulate bone grafts and/or bone substitutes over a defect before the primary closure to control tissue growth. This regeneration concept has been used for over 40 years and aims to allow cell neoformation of the desired tissue, fill a space, and prevent the growth of other undesirable cell types [1].

For GBR to be successful, the membrane must remain in place for a period of time, allowing the repair compartment to be populated by osteoprogenitor cells and the volume of space to be regenerated. The receptive area, as well as the flap, can promote angiogenesis permeating the membrane. In contrast, osteoblasts synthesize bone matrix only in the vicinity of blood vessels, and the change in oxygen tension can alter cell gene expression to fibrous tissue and fibrocartilage, thus ensuring ample blood support and mechanical support [2,3].

The bone vasculature plays an important role in the angiogenesis process. The cell type and the systemic health of the individual interfere with homeostasis and wound healing because different angiogenic factors can be expressed. At the bone regeneration process, a higher amount of oxygen was necessary; therefore, the more organized and specific the formation of blood vessels were, the more success will be achieved [4].

Due to the process of bone healing and repair and the current need for bone reconstruction and regeneration to rehabilitate patients with implant-supported prostheses [5], studies on all the phases that make up this process have become essential to achieve success in clinical procedures. Several bone grafting techniques have advanced over the years, from the use of membranes, first described in orthopedics, improved for use in guided tissue regeneration [6], their use in guided bone regeneration (GBR) procedures has become indispensable because it acts as a barrier to cell selection [7,8].

Membranes used in GBR procedures can be absorbable or non-absorbable [9,10]. Several membranes have been tested in vivo and in vitro, either with or without biomaterials, to study their biological behavior [11,12,13]. Aspects such as degradation time, inflammation, cell occlusion, and volume maintenance are some of the biological aspects studied to ensure that a particular membrane is indicated for use according to the clinical conditions of each case [14,15]. Collagen is one of the most used components for the manufacture of absorbable membranes used in ROG due to its high biocompatibility; however, the major disadvantage is its reduced dimensional stability, and to improve this property, companies use crosslinks.

Materials developed for the health area, in many situations, have little information in their catalogs and must undergo experimental studies to verify if they meet the requirements for clinical application [5]. Therefore, for the study of absorbable membranes, we must understand their performance during all phases of bone repair. For bone formation to occur, membranes must have two indispensable prerequisites: ample blood support and mechanical support. Results have shown that angiogenesis is a determining factor in bone neoformation, due to the transport of osteoprogenitor cells and nutrients that is only possible on a stable surface [3].

In the field of tissue engineering, the development of devices, such as the use of the magnetic field, that can integrate statical materials, such as membranes, with active cells, and the production of hybrid materials that have better mechanical properties without interfering in the biocompatibility, have progressed in an attempt to improve the biological results of the absorbable membranes [16,17].

The Jason^®^ membrane (Botiss Biomaterials GmbH, Zossen, Germany) hich originates from the porcine pericardium, has a natural multilayer structure and offers an extended barrier function from four to six months. This ensures successful regeneration, especially for larger procedures. This membrane, based on type III collagen, is resistant to rupture and can be fixed with screws and sutures without breaking. Furthermore, its small thickness (0.1–0.25 mm) allows for surface adaptation and tension-free wound closure. The Jason porcine pericardium membrane acts as a long-term barrier, giving the graft material sufficient time to integrate into the receptor area [18].

On the other hand, the Collprotect^®^ membrane (Botiss Biomaterials GmbH, Zossen, Germany) is produced from natural collagen, and its maintenance is guaranteed during the cleaning and elimination processes of all antigenic and non-collagenous components. It is made from the porcine dermis and has a uniform thickness of 0.4 mm. It allows for intermediate protection and has an open and porous three-dimensional organization, which ensures the growth of blood vessels and cell adhesion. In addition, it produces a natural homeostatic effect and can be handled dry or wet without the risk of self-adhesion [18].

New membranes are often marketed without prior evidence from animal research to validate and observe the characteristics of these materials. Thus, we aimed to evaluate the biological behavior and osteopromotive factor of two collagen membranes of porcine origin recently introduced in the market that do not present many studies on their biological behavior and their performance in critical situations for GBR, through comparative analyses with Bio-Gide^®^ (Geistlich, Wohlhusen, Switzerland). Bio-Gide^®^ is the leading membrane in its category and is composed of collagen types I and III that are presented in a double layer (one smooth, one porous) and are not crosslinked.

## 2. Materials and Methods

### 2.1. Study Design

In this study, an in vivo, comparative, blind, and randomized study was conducted. This study was approved by the Ethics Committee on Animal Use (CEUA) of the Araçatuba Dental School—UNESP, under protocol #01062-2017. The experimental handling procedures were performed following the standards established by the “Guide to the care and use of experimental animals” [19] in addition to the ARRIVE guidelines [20].

### 2.2. Sample

For this study, a total of 96 adult (three to four months) male rats (*Rattus norvegicus albinus*, Wistar) weighing approximately 250 g to 350 g were used. The rats were randomly divided into four groups containing 24 animals (*n* = 6 per group) using the manual drawing method and sacrificed at four experimental periods: seven, 15, 30, and 60 days after surgery. The sample size was calculated using the SigmaPlot 12.0 program (exact graphs and data analysis, Sant Rose, LA, USA). For this, we used the results of a previous study in which the minimum difference in the percentage means for bone neoformation was 18.9, with an expected standard deviation of 8.1. Five samples in each experimental group would be needed for a power test of 80% and *p* > 0.005 [21]. Therefore, considering the possibility of losing any animal during the experiment, six animals were included in each group.

These animals were kept in the vivarium of the Faculty of Dentistry of the Araçatuba Campus—UNESP in cages. Each cage blinded to the operator, contained three animals, and was cleaned every two days. The animals were fed balanced chow (NUVILAB, Curitiba, Brazil) containing 1.4% Ca and 0.8% P and water ad libitum. In each animal, a critical bone defect of 8 mm in diameter [22] in the calvaria was performed, and the defect was filled with each proposed treatment: Bio-Gide^®^ (BG) (positive control), Jason^®^ (JS), Collprotect^®^ (CS) (experimental groups), and clot (CG) (negative control).

### 2.3. Experimental Surgery

The animals were fasted preoperatively for 12 h and were sedated by intramuscular administration of ketamine hydrochloride (Francotar; Vibrac do Brasil Ltd., São Paulo, Brazil) along with xylazine (Rompum, Bayer AS, Saúde Animal, São Paulo, Brazil), at a dosage of 50 mL/kg and 0.5 mL/lg, respectively.

Trichotomy was performed in the calvaria region, and a strict aseptic protocol was adopted, including antisepsis with 10% polyvinyl pyrrolidone iodine degermant (Riodeine Degermante, Rioquímica, São José do Rio Preto, Brazil). All of the instruments and drapes used were sterilized to ensure the asepsis of the operated area.

**Figure 1:**
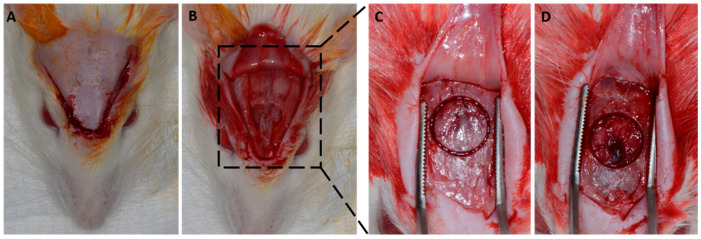
Experimental surgical procedure, performed after antisepsis in the region. (**A**) Trichotomy performed and V incision in the occiput-frontal direction; (**B**) exposed calvaria after total flap detachment; (**C**) marking of the 8 mm defect to be created; and (**D**) 8 mm critical defect performed while preserving the dura mater.

**Figure 2:**
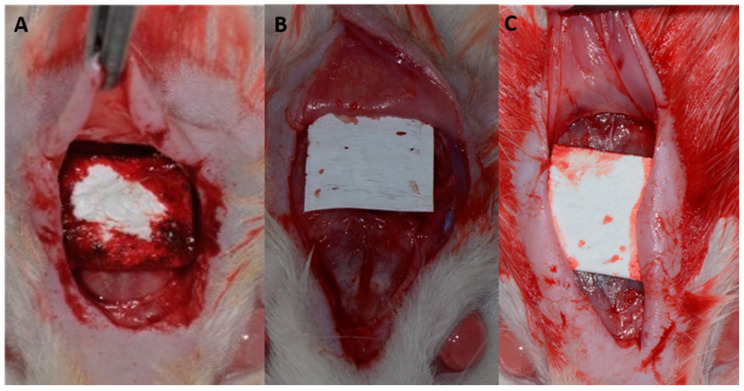
(**A**) Bio-Gide^®^ porcine collagen membrane fitted over the bone defect; (**B**) Jason^®^ porcine dermis collagen membrane adapted over the bone defect; and (**C**) Collprotect^®^ porcine pericardium collagen membrane adapted over the bone defect.

A v-shaped occipitofrontal incision of 2 cm was made with total flap detachment. A critical defect of 8 mm was created in the central portion of the calvaria (Figure 1). According to the proposed treatments, the defects were filled with blood clots, and different membranes were positioned (Figure 2).

At the end of the procedure, the soft tissues were carefully repositioned and sutured in the planes. In the immediate postoperative period, each animal received a single intramuscular dose of 0.2 mL of penicillin G benzathine (Pentabiotic Veterinário Pequeno, Fort Dodge Saúde Animal Ltd., Campinas, São Paulo, Brazil). The animals were kept in individual cages throughout the experiment with food and water ad libitum and were taken out every two days.

### 2.4. Histotechnical Processing and Computerized Microtomography (Micro-CT)

The rat calvaria, obtained at seven, 15, and 30 days after the animals were sacrificed, were removed and fixed in 10% formaldehyde solution for 48 h, washed in running water for 24 h, decalcified in 20% ethylenediaminetetraacetic acid for five weeks, dehydrated in a sequence of alcohols, and diaphanized. Subsequently, the calvaria was cut in half in the longitudinal direction to separate the bone defects. The pieces obtained were embedded in paraffin and cut into 6 μm thick semi-serial cuts. Ten slides were obtained from each piece, which were stained with hematoxylin and eosin (H&E) for the preparation of histological and histomorphometric analyses. Only in the 60-day experimental period were the pieces analyzed using the micro-CT system Skyscan 1174 (Bruker, Kontich, Belgium), after being fixed in 10% formaldehyde for 48 h, washed in running water for 24 h, and kept in 70% alcohol. These pieces were then treated the same as the others, and the slides were stained with H&E.

### 2.5. Histological and Histomorphometric Analysis

Before performing the histometric analysis, the samples were coded so that only the advisor knew the groups to which they belonged. A single examiner performed the analyses.

After obtaining the slides, an optical microscope (LeicaR DMLB, Heerbrugg, Switzerland) coupled to a camera (LeicaR DC 300F microsystems Ltd., Heerbrugg, Switzerland) and connected to a computer with ImageJ software (National Institutes of Health, Bethesda, MD, USA) was used to take photomicrographs to perform the histological and histomorphometric analyses.

The inflammatory profile of the membranes was determined by the quantification of inflammatory cells (lymphocytes) and blood vessels. For this, photomicrographs were taken at 100× magnification, of which three images were taken by histological section: the first, in the center of the defect, the second on the right, and the third, on the left. Two histological sections per animal were chosen from samples obtained at days seven and 15 for the JS, CS, and BG groups, totaling 72 images. After obtaining the images, a grid containing 130 points was inserted in each image using ImageJ software. Each cell with lymphocyte characteristics (mononuclear) that touched the point was counted, and the set of points inserted within the same vessel was quantified.

The amount of newly formed bone (primary outcome) was quantified from the panoramic reconstruction of the histological sections at 6.3× magnification. The ruler tool in ImageJ was used for the calibration according to the magnification chosen for histometry. The known distance was calibrated to 1, and the unit of measurement was in micrometers (µm). After calibrating the ruler, the polygon tool was used to count the area after the image was opened in the software. With the image open, the polygon tool was executed to delimit the area to be contacted, and the results were saved and added at the end of the process. This represented the amount of bone neoformation. Photomicrographs were taken close to the bone stump and in the center of the defect at 40× magnification. Using the polygon tool in ImageJ, the area of neoformed bone was dimensioned for all groups in all experimental periods.

### 2.6. Immunohistochemical Analyses

During the histotechnical process, some histological blades were separated from the semi-serial cuts for immunolabeling. Antigenic recovery was performed in a pressure cooker (Electrolux Chef), and endogenous peroxidase activity was blocked with a hydrogen peroxide solution diluted in methanol for 15 min. Subsequently, the tissue was blocked with 3% bovine serum albumin (BSA) diluted inTBS-T (1% Triton X-100, 100 mM of Tris, pH 7.4. In the next step, the sections were subjected to reaction with specific primary antibodies against osteocalcin (Santa Cruz Biotechnology, Dallas, TX, USA) and osteopontin (Santa Cruz Biotechnology, Dallas, TX, USA) and incubated in a humid chamber overnight. The following day, the sections were incubated with a polyclonal biotinylated secondary goat antibody produced in donkeys (Jackson ImmunoResearchLaboratories, West Grove, PA, USA) at room temperature, developed with diaminobenzidine (DAB), and stained with Harris hematoxylin. Furthermore, the end of the reaction was carried out against the cut staining with the Harris hematoxylin. For each antibody, the immunolabeling intensity of the relevant proteins was assessed semi-quantitatively by assigning different scores according to the number of cells immunolabeled in the bone repair process, and analysis was performed using the aforementioned R DMLB light microscope. Immunolabeling intensity was scored from 1 to 4, with 1 being the absence of immunostaining and 4 being intense labeling.

### 2.7. Micro-CT Analysis

The parameters used were: pixel size 11.87 µm, 50 kVp, 0.5 mm aluminum filter, 0.6° rotation, and 180° arc rotation. After digitization, the images obtained were imported into the NRecon Reconstruction Software (Skyscan, Bruker, Kontich, Belgium) for three-dimensional (3D) reconstruction of the calvaria on a grayscale. After obtaining the 3D images, DataViewer software (Skyscan, Bruker, Kontich, Belgium) was used to determine the volume of interest, which was standardized for all images, all of which were saved in a coronal view.

The cuts obtained were imported into CT-Analyzer software (Skyscan, Bruker, Kontich, Belgium). In the images obtained, morphometric parameters were evaluated, such as bone volume (BV), percentage of bone volume (BV/TV), trabecular bone thickness (Tb.th), number of bone trabeculae (Tb.N), trabecular meshwork (Tb.Sp), and percentage of total bone porosity (Po.tot) (Skyscan, Bruker, Kontich, Belgium). The round tool was used to determine the region of interest due to the rounded morphology of the defects, which was also standardized for all reconstructions (9.74 × 9.74). Soon after, a grayscale range of 105 to 242 in 40 layers was used. The images were then converted to grayscale to calculate the three-dimensional parameters in millimeters (mm) using the software.

### 2.8. Statistical Analysis

Data obtained during histomorphometric and micro-CT analyses were subjected to a normality test to assess the distribution of samples (Shapiro–Wilk, *p* > 0.05). After confirming the normal distribution of the samples, for histomorphometric analyses, two-factor analysis of variance (ANOVA) and Tukey post-test were used to compare the means obtained. For the micro-CT analyses, one-factor ANOVA and Holm–Sidak post-test were used to compare the means obtained. A *p*-value of <0.05 was considered statistically significant.

## 3. Results

Among the samples, no complications were observed during the surgical procedure, and all animals survived the postoperative period.

### 3.1. Histological Analysis

Histological description was performed from panoramic reconstructions of photomicrographs performed at a 6.3× magnification and confirmation of the structures found at higher magnifications.

**Figure 3:**
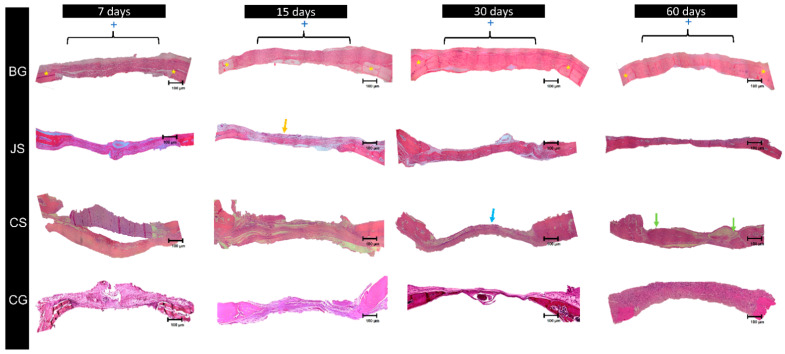
Panoramic reconstructions of the photomicrographs of the BG, JS, CS, and CG groups at all experimental times at 6.3× magnification. The 8 mm defect is delimited by the [ ] and indicated by the + symbol, the yellow * indicates the location of the bony stumps.

At seven days of bone repair, organized and highly vascularized granulation tissue was observed in the BG, JS, and CG groups. Furthermore, the CS group was also highly cellularized. At 15 days, the membranes remained intact in the BG and CS groups, which was not observed in the JS group (yellow arrow). At 30 days, bone neoformation was observed in the BG and JS groups, whereas in the CS group, little or no new bone tissue was observed in the center of the defect (blue arrow). At 60 days, in the BG group, complete closure of the defect was observed, and some specimens from the JS group also showed this characteristic. The CS groups presented more intense bone neoformation only close to the bone stumps, similar to that of the CG group (green arrow) (Figure 3).

### 3.2. Histometric Analysis

The results obtained were interpreted to trace the inflammatory profile and assess the osteopromotive capacity of each membrane compared to the positive control (BG).

#### 3.2.1. Inflammatory Profile

**Figure 4:**
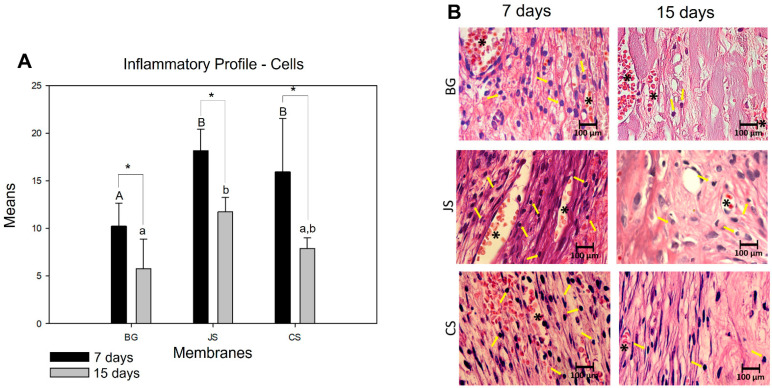
(**A**) Graph showing the comparisons of the means and standard deviations within and between groups for the analysis of the inflammatory cells (lymphocytes) at seven and 15 days. The capital letters represent the statistically significant differences between groups at seven days and the lower case letters at 15 days. The * demonstrates if there was a statistically significant difference between groups at seven and 15 days. (**B**) In the photomicrographs on the side taken at 100× magnification, the yellow arrows indicate the cells (lymphocytes) and the * blood vessels.

In an intragroup comparative analysis of the number of lymphocytes from seven to 15 days, a statistically significant difference was found in the BG (*p* = 0.027), JS (*p* < 0.001), and CS groups (*p* < 0.001). In the comparative analysis between groups at seven days, the BG group had the lowest cellular lymphocyte content, which was statistically different for the JS (*p* < 0.001) and CS (*p* = 0.024) groups. Despite having the largest amount of cellular content, the JS group did not show a statistically significant difference in the CS group (*p* = 0.691). When analyzing the evolution of the repair process at 15 days, the JS group, despite showing a milder inflammatory response, continued to show statistical differences from the BG group (*p* = 0.021). The BG and CS groups showed a significant decrease in cell content. However, the CS group continued to show no statistical difference in the JS group (*p* = 0.115) (Figure 4A,B).

**Figure 5:**
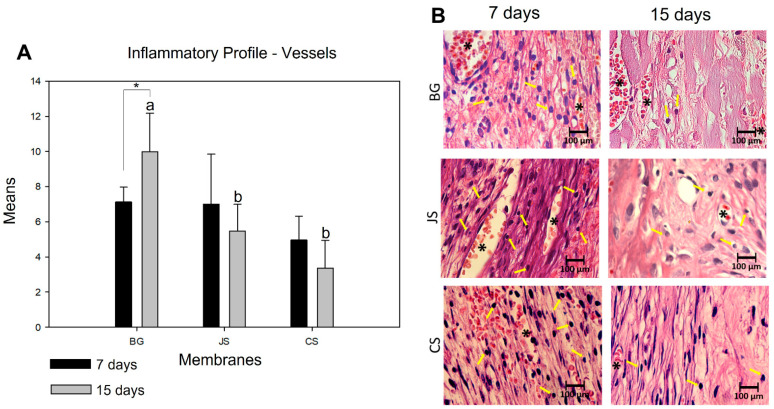
(**A**) Graph showing the comparisons of the means and standard deviations within and between groups to analyze the number of blood vessels at seven and 15 days. The lowercase letters show a statistically significant difference at 15 days. There was no statistical difference for the seven-day period. The * demonstrates whether there was a statistically significant difference within the group at seven and 15 days. (**B**) In the photomicrographs on the side taken at 100× magnification, the yellow arrows indicate the cells (lymphocytes) and the * blood vessels.

In evaluating the angiogenic capacity of the membranes, in an intragroup comparative analysis, only the BG groups (*p* = 0.029) presented a statistical difference during the evolution of the repair process from seven to 15 days. In addition to not separating statistical differences, the other groups demonstrated a decrease in the number of blood vessels. In the intergroup analysis, no statistically significant difference was observed despite the similarity in behavior between the BG and JS groups at seven days. The CS group had a lower capacity to promote the formation of new blood vessels. In the second postoperative period, the BG group showed a statistically significant difference in the CS (*p* < 0.001) and JS (*p* = 0.008) groups. In the CS group, the greatest decrease in the number of blood vessels was observed (Figure 5A,B).

#### 3.2.2. Newly Formed Bone (NFB)

In a comparative analysis of the membrane factor, only the comparison between the BG × JS (*p* = 0.990) and CS × CG (*p* = 0.345) groups did not show any statistical difference, suggesting a similar behavior between the groups. The time factor, in turn, did not influence the result, showing no statistical difference only in the comparison between days seven and 15 (*p* = 0.633).

At seven days of bone repair, all groups behaved similarly in terms of bone neoformation, with no statistical difference. At 15 days, no statistical difference was observed despite a greater amount of neoformed bone observed in the samples from the BG and JS groups.

**Figure 6:**
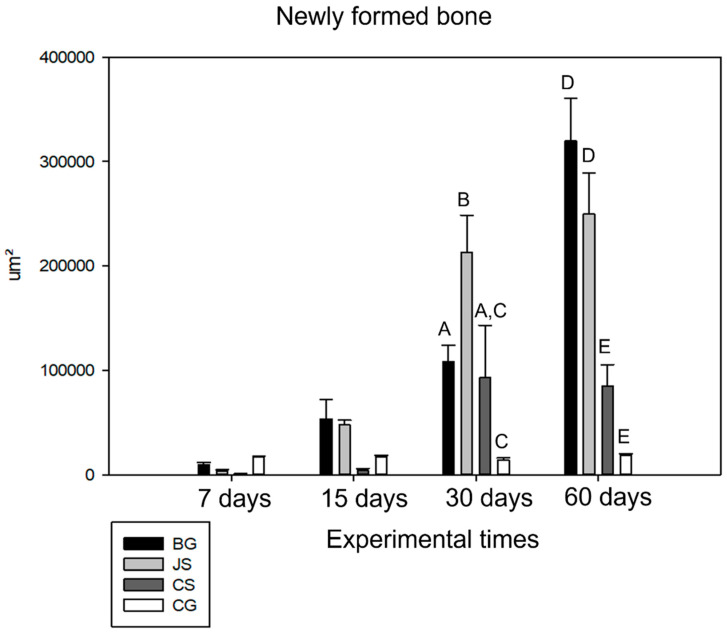
Comparative graph of the means and standard deviation of the neoformed bone area of all groups (BG, JS, CS, and CG) for the experimental times of 7, 15, 30, and 60 days. Capital letters show a statistically significant difference between groups for the periods of 30 and 60 days, in the periods of 7 and 15 days, there was no statistical difference between the groups analyzed.

At 30 days, in the later period of the bone repair process, the CG group continued to show the expected performance, and the JS group showed the most significant increase in the amount of neoformed bone, with a significant difference found for the BG (*p* = 0.009) and CS groups (*p* = 0.035). There were no statistically significant differences between the BG and CS groups. At 60 days, the BG group presented the best osteopromotive potential. However, no statistical difference was found between the BG and JS groups. The CS group, compared to the other two test groups, had the worst result during the repair process, showing no statistical difference to the negative CG and presenting a statistical difference to the JS (*p* < 0.001) and BG groups (*p* < 0.001) (Figure 6).

### 3.3. Immunohistochemical Analysis

#### 3.3.1. Osteocalcin

**Figure 7:**
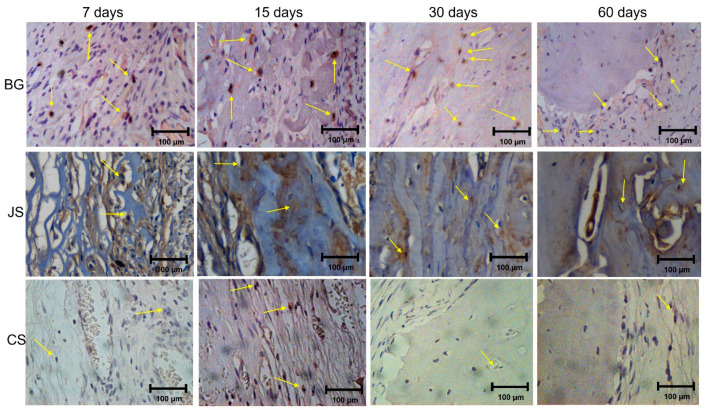
Photomicrographs of the immunohistochemical analyses at BG, JS, and CS group in 7, 15, 30, and 60 days highlight osteocalcin reaction at 40.0× magnification. The yellows arrow indicates the labeling.

For the semi-quantitative comparison, the BG group demonstrated moderate (2) labeling for OC and the JS and CS group demonstrated mild (1) labeling at 7 days. At 15 days, the test groups continued to demonstrate mild (1) while the BG group demonstrated a transition for the moderate (2) to intense (3) labeling. At 30 and 60 days, the JS group showed moderate (2) labeling and the CS group mild (1) labeling. The only group that demonstrated intense (3) labeling was the BG group (Figure 7).

#### 3.3.2. Osteopontin

**Figure 8:**
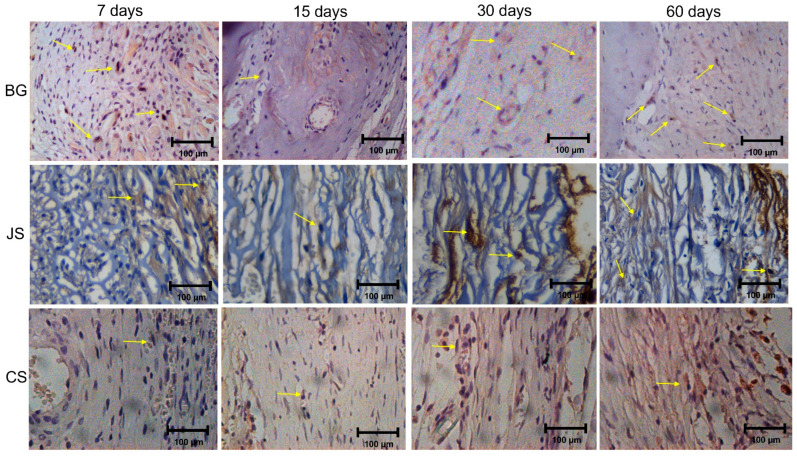
Photomicrographs of the immunohistochemical analyses at BG, JS, and CS group in 7, 15, 30, and 60 days highlight osteopontin reaction at 40.0× magnification. The yellows arrow indicates the labeling.

For the semi-quantitative comparison, the BG group demonstrated moderate (2) labeling for OC and the JS and CS group demonstrated mild (1) labeling at 7 days. At 15 days, all groups demonstrated mild (1) labeling. At 30 and 60 days, the JS and BG group showed moderate (2) labeling and the CS group mild (1) (Figure 8).

### 3.4. Micro-CT Analysis

**Figure 9:**
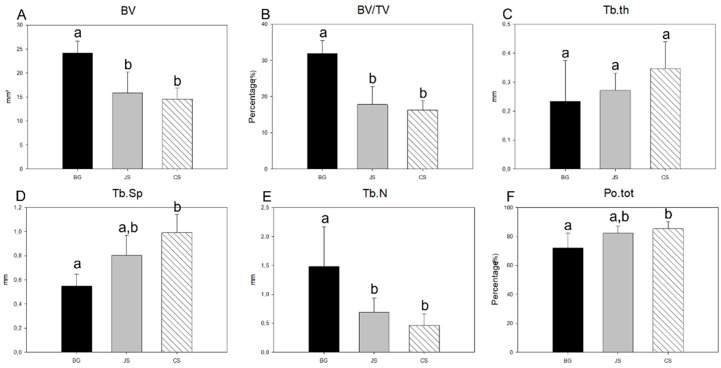
Graphs showing the mean and standard deviation of (**A**) BV, (**B**) BV/TV, (**C**) Tb.th, (**D**) Tb.Sp, (**E**) Tb.N, (**F**) Po.tot at 60 days for the BG, JS, and CS groups. Statistically significant differences between the groups are represented by the different lowercase letters (a, b, and c).

**Figure 10:**
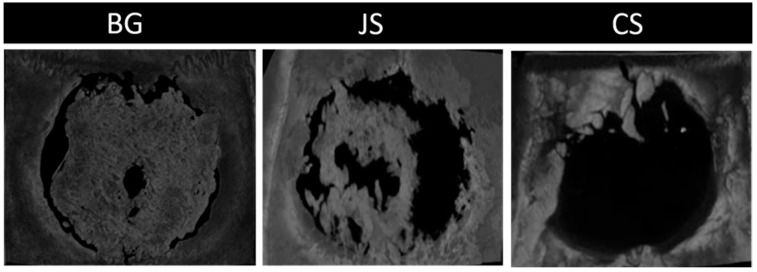
Axial reconstruction of the critical calvaria defect after 60 days of bone repair for the BG, JS, and CS groups. Note that the largest area of bone neoformation was for the BG group, followed by the JS and CS groups.

The BG group had the highest mean BV (24,127 mm^3^), followed by the JS group (15,807 mm^3^), with the CS group having the worst result (14,548 mm^3^), followed by the averages of 34.9%, 17.8%, and 16.2%, respectively, for BV/TV. In the intergroup comparative analysis, the BG group showed a statistically significant difference for the two parameters (BV and BV/TV) compared to the JS (*p* = 0.012 and *p* < 0.001) and CS groups (*p* = 0.009 and *p* < 0.001) (Figure 9A,B and Figure 10).

Concerning the bone trabeculae, the mean Tb.th of the groups was very similar between the BG (0.233 mm), JS (0.271 mm), and CS groups (0.346 mm). While the CS group had the highest mean value, no statistical difference was observed between the groups (Figure 9C). The Tb.Sp and Tb.N demonstrated the superiority of the BG group, which showed the smallest spacing of the bone trabeculae (0.548 mm) and the largest number of trabeculae (1.479 per mm), with a statistical difference in Tb.Sp only in the CS groups (*p* = 0.006) and in Tb.N for groups JS (*p* = 0.020) and CS (*p* = 0.008). For the JS and CS groups, the Tb.Sp values (0.804 mm and 0.991 mm) had similar mean values, with no statistically significant difference. For the Tb.N parameter, the JS (0.686 per mm) and CS groups (0.460 per mm) presented similar mean values with no statistically significant difference (Figure 9D,E).

Consequently, Po.tot showed the lowest porosity for the BG group (71.975%), followed by the JS group (82.204%), with no statistically significant difference. Finally, with the highest total porosity, the CS group (85.207%) showed a statistically significant difference compared to the BG group (*p* = 0.041) (Figure 9F).

## 4. Discussion

The results demonstrated that angiogenesis is directly linked to a more controlled inflammatory reaction and that around large blood vessels there is a high concentration of perivascular cells with osteopromotive potential [23]. Extensive blood support is related to the presence of nutrients, and the arrival of undifferentiated mesenchymal cells that differentiate into osteoblasts is responsible for the synthesis of the bone matrix [24]. However, in addition to the fact that the formation of organized bone tissue requires a stable mechanical surface, osteoblasts synthesize bone matrix only close to blood vessels, and the reduction in oxygen tension can genetically change these cells into cells that form fibrous tissue [3].

However, crosslinking, such as the formation of inter-molecular or intra-molecular crosslinks by ultraviolet light, glutaraldehyde, or hexamethylene diisocyanate, mineralization, and multilayer membranes, although shown to be more efficient in inhibiting the proliferation of cells such as fibroblasts, promotes greater local inflammatory reactions that interfere with the biocompatibility and inflammatory response of this membrane in the initial periods of bone repair [25,26].

The BG membrane was used as a positive control because of its high performance, as described in the literature. Moreover, BG is considered the best membrane currently available on the market [27]. In the analysis of its inflammatory profile, we were able to verify the reason for its good clinical performance. The ability to allow the entry of nutrients through angiogenesis, characterized by an increase in blood vessels and having a low inflammatory reaction for a very short period from a cellular point of view, gives this membrane an advantage over the other membranes.

When comparing the BG and JS groups, it was verified that the BG membrane has a double layer with a compact outer layer and a porous inner layer [26,27,28,29]. Moreover, the JS membrane that consists of porcine pericardium has fiber structure collagen braids that are differently oriented, which according to the manufacturer, may allow greater cell permeability. However, this high permeability was not exclusive to undifferentiated mesenchymal cells but also the presence of many inflammatory cells, particularly leukocytes. In this regard, we believe that this type of treatment for collagen stabilization may have directly influenced the type of inflammatory response. In the current study, there was a statistically significant difference in the presence of inflammatory cells at 15 days, with the number of cells being greater in the membrane of the porcine pericardium than in the BG group. This was also the conclusion reached by Rothamel et al. [26], that is, the use of porcine collagen types I and III that do not undergo crosslinked processes allowed for better tissue formation and were more vascularized, without the presence of foreign body-like reactions.

The porcine pericardium membrane (Jason^®^) is half the thickness and three times less dense than the porcine collagen membrane (Bio-Gide^®^, Geistlich Wohlhusen, Switzerland). Thus, it is assumed that its vascular supply should be higher. However, we found less formation of blood vessels in the JS group than in the BG group. This fact contrasts with the high porosity described in the product catalog and study by Ortolani et al. [18]. When analyzed in detail during the experimental period, it was observed that the difference between the two groups at seven days was not statistically significant. However, at 15 days, there were more vessels in the BG group than in the JS group. Furthermore, in the JS group, the number of vessels was smaller at 15 days than at seven days, making the vascular permeability lower, unlike what happens with BG. Therefore, it appears that both the inflammatory profile and vascular supply possibly interfere with the amount of bone tissue neoformation, as described by Patino et al. [3]. One hypothesis is that the degradation of the porcine pericardium membrane causes an inflammatory reaction greater than that of the porcine collagen membrane. At this stage, these two events become crucial for the final volume of neoformed bone tissue. This fact was confirmed when we analyzed the results obtained after 60 days.

Although 30- and 60-days specimens from the JS group presented satisfactory bone neoformation, it is important to remember that the collapse of the membrane, observed in almost all specimens, culminated in a thinner neoformed bone tissue than the BG group (Figure 6). This was expected after the mechanical studies by Ortolani et al. [18] demonstrated a tensile strength and thickness smaller than that of the BG group. The membrane derived from porcine dermis collagen (Collprotect Straumann^®^) was also evaluated in this study. This membrane is obtained from the extraction of the porcine dermis and is formed by collagen types I and III, with a rough and porous structure and a thickness of 0.4 mm. Although the membrane does not undergo a crosslinking process, structural and chemical changes occur during the manufacture of this barrier. Due to these various processes, the properties of collagen can be modified so that the material meets the requirements for specific applications such as biological barriers [18].

Significant differences were observed when analyzing the inflammatory profile of the CS group compared to the BG group. At seven and 15 days, there were significantly more inflammatory cells in the CS group. There was a decrease in cells in the intragroup comparison, with the results indicating lower biocompatibility of the membrane, which is a necessary characteristic to obtain clinical success in ROG procedures. Dupoirieux et al. [29] indicated that the membrane absorption process could have induced the inflammatory reaction [30]. However, Ge et al. [31] observed better bone regeneration using the BG membrane, which also underwent the absorption process and presented excellent clinical results. Similarly, when the vessels were analyzed at seven and 15 days, smaller vessel formation was observed in the CS group than in the BG group, even when the CS was thinner than the BG. In the intragroup analysis, the BG group showed an increase in angiogenesis, while the CS group showed a decrease, indicating lower angiogenesis, characteristics that are also essential for the effectiveness of ROG [32].

Analyzing the performance of membranes in bone neoformation, little or no bone neoformation was found at seven days, a result already expected due to the early time of the analysis. In groups BG, JS, and CS, membranes with characteristic integrity were noted in many specimens. At 15 days, the BG and JS groups demonstrated better osteopromotive potential [33].

At 30 days, the JS group showed its peak of action in the bone repair process, presenting the largest amount of neoformed bone, whereas the CS and BG groups showed similar values with a slight advantage over the BG group. The CG group performed as expected, with little or no bone neoformation, thus confirming the achievement of a critical defect [21]. At the end of the repair process, as can be seen in the histological and microtomography results, the BG group surpassed the JS and CS groups, demonstrating that its biological activity occurs during the final stages of the repair process. This finding confirmed that this membrane has slow absorption and remains in the surgical bed for the necessary period. Despite promoting bone neoformation, the JS group had results very similar to those at 30 days at 60 days, indicating stabilization of the action of the membrane in the defect. In turn, the CS group presented an unsatisfactory result, being able to promote important bone neoformation only close to the bone stumps [34,35,36].

Regarding the quality of the newly formed bone, greater porosity, greater trabecular separation, and fewer trabeculae, that is, less dense bone, were found in the CS group. Moreover, the CS group presented more vascular bone tissue that was less resistant due to its small osteopromotive capacity, which may have enabled the growth of fibrous tissue intermingled with bone tissue [5]. In contrast, the BG group presented the largest BV and the smallest porosity, characterizing a more compact bone tissue. This is an important finding as this regenerated bone tissue is intended to provide mechanical support for implant-supported prostheses [36]. The JS group presented intermediate results regarding the separation of the trabeculae and their thickness, but in much smaller numbers than the BG group, demonstrating that in critical defects and the CS group, these membranes allowed the growth of fibrous tissue intermingled with the tissue. Moreover, the newly formed bone tissue had a smaller thickness [37].

## 5. Conclusions

Given the results presented, we can conclude that despite the two membranes tested being made of collagen, their biological performance and ability to act on bone neoformation were different, reiterating the need for further in vivo studies. The JS membrane showed a satisfactory result, while the CS membrane proved ineffective in regenerative procedures, showing no statistical difference with the negative control group. Therefore, based on data present in this study, the JS membrane demonstrates to be able for clinical use in GBR techniques, although the BG membrane remains the choice for critical-size defects treatment.

## Data Availability

Not applicable.
